# Protective effects of L-arabinose in high-carbohydrate, high-fat diet-induced metabolic syndrome in rats

**DOI:** 10.3402/fnr.v59.28886

**Published:** 2015-12-10

**Authors:** Lei Hao, Xiaoling Lu, Min Sun, Kai Li, Lingmin Shen, Tao Wu

**Affiliations:** Key Laboratory of Food Nutrition and Safety, Ministry of Education, College of Food Engineering and Biotechnology, Tianjin University of Science & Technology, Tianjin, China

**Keywords:** L-Arabinose, metabolic syndrome, hypertension, insulin resistance, adipocytokines

## Abstract

**Background:**

L-Arabinose is a non-caloric sugar, which could affect glucose and lipid metabolism and suppress obesity. However, few reports have described the effect of L-arabinose in metabolic syndrome, a combination of medical disorders that increase the risk of diabetes and cardiovascular disease.

**Objective:**

This study was conducted to explore the effects of L-arabinose in rats with metabolic syndrome induced by a high-carbohydrate, high-fat (HCHF) diet.

**Methods:**

After the rat model for metabolic syndrome was successfully established, L-arabinose was administrated by oral gavage for 6 weeks. The biochemical index and histological analysis were measured, and the expression levels of genes related to fatty acid metabolism were analyzed using real-time PCR.

**Results:**

Following treatment with L-arabinose, metabolic syndrome rats had an obvious reduction in body weight, systolic blood pressure, diastolic blood pressure, fasting blood glucose, triglycerides, total cholesterol, serum insulin, TNF-α, and leptin. Further study showed that treatment with L-arabinose significantly increased the expression of mRNA for hepatic CPT-1α and PDK4, but the expression of mRNA for hepatic ACCα was reduced.

**Conclusions:**

This work suggests that L-arabinose could lower body weight, Lee's index, and visceral index and improve dyslipidemia, insulin resistance, inflammation, and viscera function, which indicate that it might be a promising candidate for therapies combating metabolic syndrome.

Metabolic syndrome is defined as a cluster of abdominal obesity, hyperglycemia, hypertension, hypertriglyceridemia, hypo high-density lipoprotein cholesterolemia, and insulin resistance (1, 2). This syndrome has become a significant public health problem worldwide because it is associated with an increased risk for stroke, type-2 diabetes, arteriosclerosis, myocardial infarction, and chronic kidney disease (3, 4). The change of dietary habits, principally the increased intake of high-carbohydrate, high-fat (HCHF) diet, which is rich in saturated fat and refined sugar, is one of the most important causes that contribute to the growing prevalence of metabolic syndrome, obesity, and type-2 diabetes (5). It is also believed that the HCHF diet contributes to cognitive decline during aging, chronic stress, and sleep deprivation and can accelerate the course of dementia in Alzheimer's disease (6, 7).

Metabolic syndrome is widely considered as a low-grade inflammatory state including inflammatory cytokine (TNF-α) and the chemokines (leptin and adiponectin) (8). Glucose and lipid-lowering drugs and insulin-sensitizing drugs have been widely used in the treatment of metabolic syndrome. However, due to their high prices and severe side effects, there is an urgent need to find some naturally occurring materials to improve treatment (9).

L-Arabinose, a monosaccharide extracted from plant gums, corn fiber, and beet pulps, is known to suppress obesity by regulating the fasting blood glucose level and the insulin resistance index (10). L-Arabinose has a very similar taste to sucrose, but with half the sweetness of sucrose; furthermore, it is not involved in metabolic processes in animals, thus L-arabinose is a non-caloric sugar. Therefore, it has shown great merits as a sweetener and a food additive to improve obesity and maintain good health. However, L-arabinose is rarely used and the physiological effects *in vivo* have not received attention. Up to now, few attempts have been made to explore the protective effect of L-arabinose in metabolic syndrome treatment. The purpose of this study was to investigate the effects of L-arabinose on hypertension, insulin resistance, and adipocytokines in rats with metabolic syndrome induced by a HCHF diet.

## Materials and methods

### Materials

Commercial kits for the measurement of alanine aminotransferase (ALT), aspartate aminotransferase (AST), free fatty acids (FFA), triglycerides (TG), total cholesterol (TC), high-density lipoprotein cholesterol (HDL-C) and low-density lipoprotein cholesterol (LDL-C), and hepatic glycogen were obtained from the Jian Cheng Biotechnology Company (Nanjing, China). ELISA kits including urea nitrogen (BUN), creatine kinase (CK), glucose transporter protein 4 (GLUT4), adiponectin (ADP), leptin, and inflammatory cytokine (TNF-α) were bought from R&D Systems Inc. (Minneapolis, USA). TRIzol reagent and the Superscript First Strand Synthesis System were purchased from Invitrogen Corp (Camarillo, USA). Glucose was acquired from TEDA Letai Chemical Co., Ltd (Tianjin, China).

L-Arabinose, which was >99% pure, supplemented in the rat's diet, was purchased from Tang He Tang Biological Science and Technology Co., Ltd. Insulin was obtained from Lilly (France). All other reagents were of analytical grade.

The blood pressure meter was obtained from Ruan Long Biological Technology Company (Beijing, China). Enzyme standard instrument was purchased from Thermo Company (Waltham, USA). Blood glucose meter was obtained from J&J Company (Neenah, USA). Real-time fluorescent quantitative PCR meter was provided by Bio-Rad (Hercules, USA).

### Animals and diets

A total of 56 weaning male Wistar rats (age: 8 weeks old; weight: 130±10 g) were purchased from Experimental Animal Center of Beijing University. All animals were individually housed and maintained in an environmentally controlled breeding room (temperature: 24±2°C; humidity: 50±10%; 12 h light/dark cycle). Animal maintenance and handling were performed according to the National Institutes of Health's ‘Guide for the Care and Use of Laboratory Animals’. Rats had unrestricted access to food and water, and the protocol was approved by the Institutional Animal Ethics Committee of Beijing Lab animal management office (license number: SCXK2011-0012, validation date: Feb 27th, 2013). Rats were adapted to the commercial pellet feed for a week and were then divided into two groups: the control group (Control, n=24), which were fed with a standard normal diet with normal drinking water; and the metabolic syndrome group (Model, n=32), which were fed with a HCHF diet for 14 weeks to induce metabolic syndrome. The composition of the HCHF diet followed the method of Poudyal and co-workers (11).

After 14 weeks, the metabolic syndrome group rats showed characteristics of abdominal obesity, hypertension, insulin resistance, and lipid metabolic disorders, indicating that the rat model for metabolic syndrome was successfully established. A total of 24 metabolic syndrome rats were selected for further study. All of the rats were randomly divided into four groups according to the following protection: Control Group (Control, n=12): normal rats were fed with normal chows, and distilled water was administrated by gavage for the following 6 weeks; Control+L-arabinose Group (Control+LA, n=12): normal rats were fed with normal chows, and L-arabinose (400 mg/kg bodyweight/day) was administrated by gavage for the following 6 weeks; Model Group (Model, n=12): metabolic syndrome rats were fed with the HCHF diet, and distilled water was administrated by gavage for the following 6 weeks; Model+L-arabinose Group (Model+LA, n=12): model rats were fed with the HCHF diet, and L-arabinose (400 mg/kg bodyweight/day) was administrated by gavage for the following 6 weeks. Rats were monitored daily for body weight and food and water intake.

### Sample collection and preparation

At the end of each treatment period, blood samples were collected by retro-orbital sinus puncture. Serum was separated by centrifugation at 3,000×*g* for 15 min at 4°C and stored at −80°C for further analysis.

At the end of the experimental diet period, the rats were fasted for 12 h and were sacrificed under carbon dioxide anesthesia. Before being sacrificed, body weight and body length were measured, and Lee's index was calculated as body weight (g)^1/3^×1,000/length (cm). Blood samples were collected from the femoral artery into 1.5 mL EP tubes, centrifuged at 3,000×*g* for 15 min at 4°C, and then stored at −80°C for biochemical analysis.

Livers were rapidly dissected and weighted to determine the liver index (liver index (%)=liver weight (g)/body weight (g)×100). Each liver was divided into three parts. One part was cut and fixed in 10% (v/v) neutral formalin solution for histological analysis. Another part was used for biochemical analysis and the remaining part was used for molecular detection. Furthermore, all visceral adipose tissues, including perirenal, retroperitoneal, and epididymal fat pad, were carefully removed and used to calculate the adiposity index (adiposity index (%)=visceral adipose tissues weight (g)/body weight (g)×100). One part of epididymal white adipose tissue was cut and fixed in 10% (v/v) neutral formalin solution for histological analysis, and the remaining part was used for molecular detection.

Skeletal muscle and liver were rapidly dissected and preserved at −80°C, which was kept for measurement of GLUT4, liver TG, and liver TC.

### Systolic blood pressure and diastolic blood pressure measurement

The rat's systolic blood pressure (SBP) and diastolic blood pressure (DBP) were measured by a non-invasive blood pressure meter every 2 weeks. The blood pressure value used for analysis was the mean of five measurements.

### Biochemical analysis

#### The analysis of blood glucose parameters

Blood glucose taken from rats’ tails (after fasting for 10 h) was measured by blood glucose meters every 2 weeks. Serum insulin concentration and HbA1c levels were determined using commercially available ELISA kits according to the manufacturer's instructions. The skeletal muscle homogenate prepared in 10% potassium chloride solution chilled on ice was used to measure the activity of GLUT4. Hepatic glycogen levels in the liver were measured using a commercial kit.

#### The analysis of lipid profile

Serum levels of TG, TC, LDL-C, HDL-C, and FFA were measured using commercial kits according to the manufacturer-provided standards and protocols. The liver tissue homogenate in appropriate dilutions was used to determine levels of liver TG and TC.

#### The analysis of safety parameters

The serum levels of TNF-α, the chemokines leptin and adiponectin, ALT, AST, BUN, and CK were measured using commercially available ELISA kits according to the manufacturer's instructions.

### Evaluation of insulin sensitivity

The insulin sensitivity was evaluated by insulin tolerance tests (ITTs), oral glucose tolerance tests (OGTTs), and the homeostasis model assessment of insulin resistance (HOMA-IR) index. Diet was removed for 4 h before the tests. Blood glucose concentrations were measured before and after injection of insulin (0.6 U/kg) and glucose (2 g/kg) for 30, 60, and 120 min. The area under the glucose-time curse (AUC) was also calculated by the following formula:

(AUC_0–120min_ = (G_0_+G_30_) × 30/2 + (G_30_+G_60_) × 30/2 + (G_60_+G_120_) × 60/2)

HOMA-IR index was determined according to the formula:

(HOMA – IR = FBG × FINS/22.5)

fasting blood glucose (FBG), fasting insulin (FINS)

### Histological analysis of liver and white adipose tissue

Small pieces of liver and epididymal white adipose tissue (about 10 g) were fixed in 10% neutral formalin solution and embedded in paraffin. Tissues were cut into slices of 3–6 µm thickness and stained with hematoxylin–eosin (H&E).

### Real-time PCR

The expression levels of mRNA in hepatic and epididymal tissue were measured by real-time PCR. For total-RNA preparation, frozen tissues were ground into fine powder in liquid nitrogen and homogenized in TRIzol solution. RNA was isolated according to the manufacturer's instructions. Total RNA was treated with RNase-free DNase and the quality and integrity were confirmed with agarose gel electrophoresis. As previously mentioned, first strand cDNA was synthesized using the PrimeScript RT reagent Kit and PCR-amplification was performed (20 µL volume) using 2 µM for each primer (Table 1), 50 ng of cDNA and 10 µL of SYBR Primix Ex-Tag. Negative controls lacking the cDNA template were included in this experiment. Thermal cycling conditions included a first denaturation step at 95°C for 3 min, and then 40 cycles performed at 95°C for 20 s, 55°C for 20 s, and 72°C for 20 min. Moreover, the GAPDH gene was used as an internal control to normalize expression, and gene expression was quantified using the 2^−ΔΔCT^ method.

**Table 1:** Sequences of the primers used in the PCR measurements

Gene	Sense	Sequence(5′-3′)
TNF-α	TNF-α-FWD	GTAGCAAACCACCAAGCG
Leptin	TNF-α-REV	GGTATGAAATGGCAAATCG
IL-6	Leptin-FWD	TTGGTCCTATCTGTCCTATGTTC
ACCα	Leptin-REV	GGAGGTCTCGCAGGTTCT
CPT-1α	IL-6-FWD	GGCTGAAAGACAATGACTAC
PDK4	IL-6-REV	TCAAGATTCCCAGAAAGAG
GAPDH	ACCα--FWD	TGGAACCAGAAGGGACAGT
	ACCα-REV	CTCAGCCAAGCGGATGTA
	CPT-1α-FWD	GCTGGGTTACTCAGAGGATG
	CPT-1α-REV	GAAAGAGTCAAATGGGAAGGA
	PDK4-FWD	AGCCCAGAAGACCAGAAAG
	PDK4-REV	CCATCGTAGGGACCACATTA
	GAPDH-FWD	GCAAGTTCAACGGCACAG
	GAPDH-REV	GCCAGTAGACTCCACGACAT

### Statistical analysis

All data were expressed as mean±standard deviation (SD). The one-way ANOVA with Duncan's multiple comparison test was performed with SPSS software, version 17.0 (SPSS Inc., Chicago, IL, USA) to assess data differences among various groups. *P*-value <0.05 was considered statistically significant. Origin 8.0 was used for figures.

## Results

### Metabolic syndrome induced by HCHF diet in rats

The metabolic syndrome model was achieved by a HCHF diet in Wistar male rats. Rats in the metabolic syndrome group had heavier body weight and higher DBP and SBP compared to the control group. The metabolic syndrome animals also exhibited a significant increase in fasting blood glucose (FBG) and fasting insulin (FINS); in addition, lipid metabolism disorders were manifested as fatty and hyperlipidemia (Table 2). As shown in Table 2, there were significant differences in parameters of serum lipid metabolism in the metabolic syndrome group, including hypertriglyceridemia, hypercholesteremia, and an obvious increase in LDL-C. These results indicate that the metabolic syndrome rat model was successfully established by feeding a HCHF diet according to the standards (12, 13).

**Table 2:** Body weight and biochemical parameters in Control group and Model group

	Control	Model
Number	24	24
Body weight (g)	376.73±12.11	438.71±22.68**
SBP (mmHg)	115.44±8.30	140.67±10.93**
DBP (mmHg)	87.99±5.09	110.66±6.74**
FBG (mmol/L)	4.58±0.45	5.81±0.42**
FINS (mU/L)	10.29±1.09	14.64±2.13**
Triglyceride (mmol/L)	1.21±0.31	2.33±0.78**
Total cholesterol (mmol/L)	1.20±0.31	3.55±1.08**
HDL-C (mmol/L)	0.68±0.13	0.63±0.17
LDL-C (mmol/L)	0.84±0.36	1.44±0.45**

All valued are mean±SD.

* *p*<0.05

** *p*<0.01 vs. Control.

### L-Arabinose lowered body weight, Lee's index, and visceral index

**Fig. 1:**
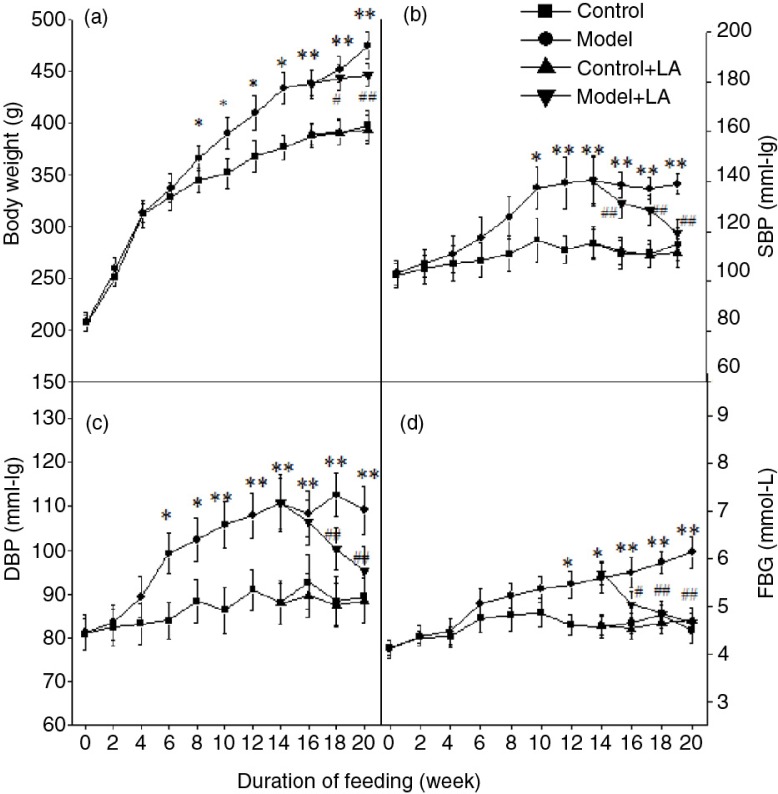
L-Arabinose administration prevented obesity, lowered SBP, DBP, and FBG in rats induced by high-carbohydrate, high-fat (HCHF) diet. All values are mean±SD. *p<0.05, **p<0.01 vs. Control; ^#^ *p*<0.05, ^##^ *p*<0.01 vs. Model.

The levels of body weight, Lee's index, and visceral index of experimental rats are shown in Fig. 1 and Table 3. Fig. 1a shows that the body weight gain in the Model group was significantly higher than that of the Control group for 8 weeks (*p*<0.05). The supplementation of L-arabinose (400 mg/kg bodyweight/day) to metabolic syndrome rats showed a remarkable decrease in body weight gain compared to the Model group (*p*<0.05) after 6 weeks of treatment. The body weight of rats in Control+LA group showed no significant variation compared to the Control group. The results indicate that L-arabinose may slow down the weight gain of obese rats. However, there was no significant difference in body length in all rats. Several other indicators, white adipose tissue weight, liver weight, liver index, and adipose index showed changes similar to those of body weight (Table 3).

**Table 3:** Effects of L-arabinose on obesity and organ weight in rats

	Control	Control+LA	Model	Model+LA
Number	12	12	12	12
Body weight (g)	407.57±30.96	402.23±27.24	485.16±32.24**	448.71±22.68^##^
Body length (cm)	21.89±0.09	21.96±0.14	22.62±0.19	22.13±0.16
Lee's index	338.03±3.48	335.47±3.24	346.68±3.67*	345.23±3.59
Liver weight (g)	9.11±0.94	8.76±0.61	13.86±1.67**	9.77±1.28^##^
White adipose tissue (g)	6.92±3.03	6.60±2.17	22.21±4.57**	16.52±3.60^##^
Liver index (%)	2.26±0.15	2.24±0.09	3.00±0.27**	2.18±0.18^##^
Adipose index (%)	1.71±0.74	1.69±0.58	4.79±0.73**	3.63±0.68^##^

All values are mean±SD.

* *p*<0.05

** *p*<0.01 vs. Control

# *p*<0.05

## *p*<0.01 vs. Model.

### L-Arabinose lowered SBP and DBP

The effect of L-arabinose on SBP in experimental animals is shown in Fig. 1b. SBP in the Model group was higher than that of the Control group after 10 weeks of treatment. However, the supplementation of L-arabinose to metabolic syndrome rats showed a significant decrease in SBP compared to the Model group during 6-week treatment (*p*<0.01). In addition, SBP of rats in Control+LA group showed no remarkable difference compared to the Control group. The results indicate that L-arabinose has an ability to decrease SBP levels in the hypertensive state. The indicator of DBP expressed similar changes to SBP.

### L-Arabinose reduced FBG and ameliorated insulin sensitivity induced by HCHF diet

The effect of L-arabinose on FBG in experimental animals is shown in Fig. 1d. After being treated with L-arabinose for 6 weeks, FBG decreased by 24.1% to a near-normal level. The FBG of rats in Control+LA had no significant change compared to the Control group. These data suggest that L-arabinose has a protective effect on hyperglycemia rats.

The effect of L-arabinose on HbA1c in experimental rats is showed in Table 4. The HbA1c levels reached 6.33±0.48 (%) for the Model group and 4.49±0.27 (%) for the Control group (*p*<0.01, respectively). After administering L-arabinose (400 mg/kg bodyweight/day) for 6 weeks, the HbA1c of metabolic syndrome rats returned to normal levels. However, the HbA1c level in Control+LA had no difference with the Control group.

**Table 4:** Effects of L-arabinose on insulin resistance and blood glucose parameters in HCHF diet-induced rats

	Control	Control+LA	Model	Model+LA
Number	12	12	12	12
Blood glucose (mmol/L)	4.49±0.30	4.19±0.31	7.21±0.59**	5.45±0.38^##^
Insulin (mmol/L)	10.74±1.29	12.19±2.18	17.07±1.98**	13.08±1.62^##^
HOMA-IR	1.93±0.29	2.04±0.39	5.46±0.65**	3.17±0.31^##^
Hepatic glycogen (mg/g)	3.35±0.39	3.15±0.42	2.61±0.56*	2.99±0.41^#^
HbA1c (%)	4.49±0.27	4.24±0.23	6.33±0.48**	5.16±0.33^#^
GLUT4 (µg/L)	53.25±5.87	58.74±7.21	44.32±6.65*	62.29±8.02^##^

All values are the mean±SD.

* *p*<0.05

** *p*<0.01 vs. Control

# *p*<0.05

## *p*<0.01 vs. Model.

Table 4 exhibits the effect of L-arabinose on GLUT4 in experimental animals. GLUT4 levels were lower in the Model group rats than that in the Control group rats, and this was normalized in the Model+LA group rats (*p*<0.05). There was no significant difference between the Control+LA group and Control group. These results suggest that L-arabinose possessed a blood glucose-lowering effect.

The rats induced by the HCHF diet developed insulin resistance, indicated by higher FBG, higher FINS and higher HOMA-IR. Compared to the Model group, insulin levels and HOMA-IR values were reduced by 23.5 and 41.9% in the Model+LA group rats, respectively. Moreover, L-arabinose did not lower the levels of blood glucose and serum insulin in the normal state (Table 4).

**Fig. 2:**
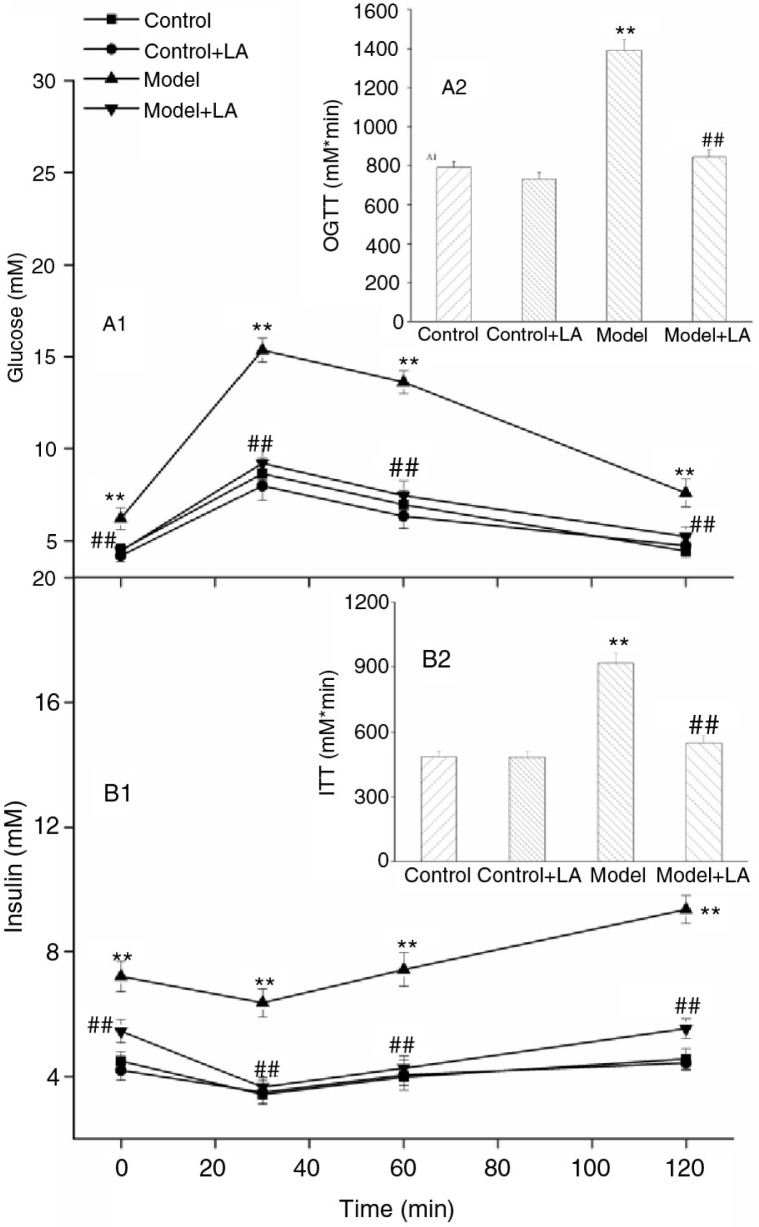
Effects of L-arabinose on oral glucose tolerance test and insulin tolerance test. Oral glucose tolerance test (A1) and insulin tolerance test (B1) performed on rats. The corresponsive AUC over 2 h was shown (A2, B2). L-Arabinose improved the glycometabolism and insulin resistance. All values are mean±SD. *p<0.05, **p<0.01 vs. Control; ^#^ *p*<0.05, ^##^ *p*<0.01 vs. Model.

In the Model group, OGTTs showed impaired glucose tolerance in comparison with the Control group; the blood glucose peak decreased slowly and the entire area under the curve increased by 76.3%. The blood glucose peak decreased by 36.6% and AUC dropped by 47.6% after the 6-week treatment with L-arabinose, which indicates that glucose resistance is regulated by L-arabinose (Fig. 2a). For ITTs, the Model group presented a mild response to extraneous insulin, which was ameliorated by L-arabinose treatment (Fig. 2b). These results suggest that L-arabinose may improve glucose tolerance and insulin sensitivity induced with a HCHF diet.

### L-Arabinose improved HCHF diet-induced lipid metabolic disorder and hepatic dysfunction

Table 5 shows the effect of L-arabinose on serum lipid profiles. The metabolic syndrome rats showed significant increases in TG, TC, and LDL-C (*p*<0.01) compared to the Control group.

**Table 5:** Effects of L-arabinose on lipid profiles and safety parameters in rats induced by HCHF diet

	Control	Control+LA	Model	Model+LA
Number	12	12	12	12
Serum TG (mmol/L)	0.75±0.27	0.56±0.20^§^	1.50±0.36**	0.96±0.34^##^
Serum TC (mmol/L)	1.57±0.38	1.48±0.27	2.51±0.42**	1.84±0.45^##^
Serum HDL-C (mmol/L)	0.73±0.22	0.89±0.29^§^	0.50±0.11**	0.59±0.16^#^
Serum LDL-C (mmol/L)	0.89±0.27	0.88±0.21	1.57±0.34**	1.18±0.33^##^
Liver TG (mmol/L)	1.09±0.27	1.12±0.24	2.98±0.65**	1.45±0.37^#^
Liver TC (mmol/L)	0.94±0.19	1.18±0.20	3.20±0.87**	1.37±0.35^##^
AST (U/L)	25.13±4.26	28.39±4.71	47.96±6.19**	30.38±5.21^##^
ALT (U/L)	16.53±3.69	15.40±3.56	38.77±5.25**	22.25±4.64^##^
ALT/AST	0.66±0.31	0.54±0.29	0.81±0.43**	0.73±0.37^#^
FFA (mmol/L)	169.85±13.72	190.11±17.49	322.50±24.57**	216.54±21.68^##^
BUN (mmol/L)	5.76±1.43	6.02±1.51	8.89±1.59**	7.26±1.77^#^
CK (mmol/L)	202.74±13.80	194.16±14.92	236.14±15.29**	223.53±16.85^#^

All values are the mean ±SD.

* *p*<0.05

** *p*<0.01 vs. Control

# *p*<0.05

## *p*<0.01 vs. Model

§ *p*<0.05 vs. Control.

The HDL-C level of metabolic syndrome rats decreased by 31.5% compared to the Control group. Whereas L-arabinose reversed the changes after administering L-arabinose (400 mg/kg bodyweight/day) for 6 weeks; the lipid metabolism profiles in the metabolic syndrome group returned to the near-normal levels. In the Control+LA group, the serum TG was significantly lower than that of the Control group, whereas serum HDL-C was significantly higher than that of the Control group (*p*<0.05). These results indicate that L-arabinose could elevate the content of serum HDL-C, reduce the content of serum TG in normal rats, and induce lipid-lowering in metabolic syndrome rats.

Serum FFA levels are shown in Table 5. The concentration of FFA in the Model group was 1.9-fold higher than the Control group. The difference changed to 1.2-fold at the end of the treatment period, which suggests that L-arabinose has an ability of lowering FFA levels in metabolic syndrome rats.

**Fig. 3:**
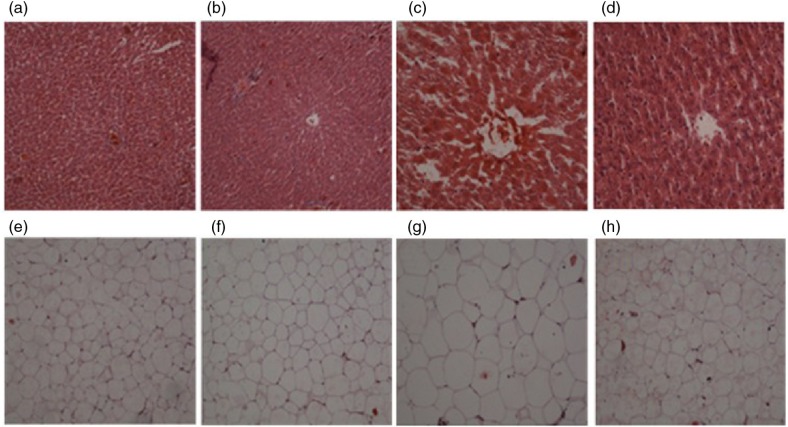
Histological sections of liver and white adipose tissue. (a) normal liver histology of Control rats; (b) normal liver histology of Control+LA rats; (c) steatosis, inflammation and, fibrosis in Model rats; (d) attenuated lipid deposition and inflammation in Model+LA rats; (e) normal WAT histology of Control rats; (f) normal WAT histology of Control+LA rats; (g) hypertrophic adipocytes in Model rats; (h) smaller adipocytes in Model+LA rats.

To determine the lipid dystopia deposition, pathological tests on the liver were performed. H&E staining revealed severe steatohepatitis in the livers from the Model group rats (Fig. 3c) compared to livers from the Control group (Fig. 3a). Administration of L-arabinose significantly attenuated steatosis, inflammation, and fibrosis (Fig. 3d). In addition, we conducted pathological tests of white adipose tissue and the sizes of adipocytes in the four groups are shown in Fig. 3e, h. The sizes of the adipocytes in the Model group (Fig. 3g) were larger than in the Control group (Fig. 3e). However, the hypertrophic adipocytes were reversed by L-arabinose treatment (Fig. 3h).

In agreement with the results from the morphological observations, the hepatic TG and TC content were increased by 1.7-fold and 2.4-fold, respectively, in the Model group compared with the Control group. After L-arabinose treatment, these values returned to near-normal levels in the Model+LA group. Moreover, the hepatic glycogen levels of the Model group decreased by 22.1% compared to the Control group. The supplementation of L-arabinose to metabolic syndrome rats reversed the change to near-normal levels (Table 5).

To further evaluate the degree of liver injury and the potential effect of L-arabinose in the metabolic syndrome model, serum ALT and AST levels were determined. As shown in Table 5, serum ALT and AST levels of the Model group rats increased by 134.5% and 90.8%, respectively. After L-arabinose treatment, the ALT and AST levels decreased significantly compared to the Model group (*p*<0.01).

### Effects of L-arabinose on safety parameters

**Fig. 4:**
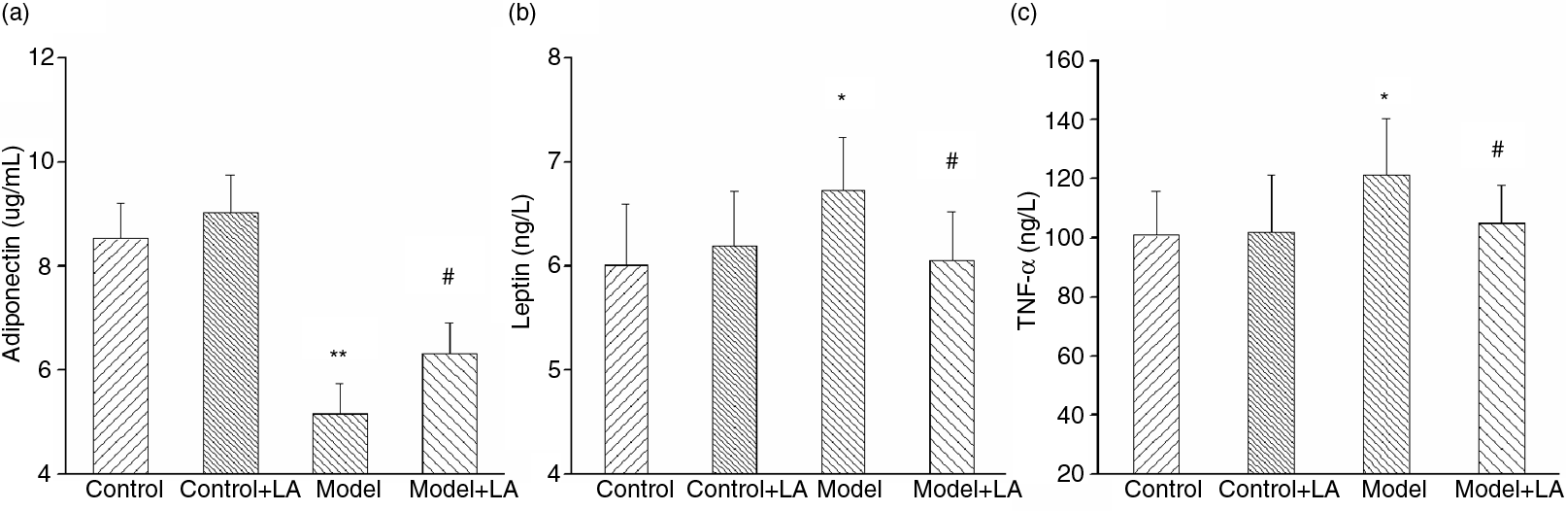
L-Arabinose lowered the levels of inflammation factors in rats induced by high nutritious diet. Adipokines (adiponectin and leptin) (a and b) and inflammatory factor (c) changed markedly, after L-arabinose treatment. All values are mean±SD. *p<0.05, **p<0.01 vs. Control; ^#^ *p*<0.05, ^##^ *p*<0.01 vs. Model.

The level of adiponectin in the Model group was decreased compared to the Control group (Fig. 4a), while leptin and TNF-α were markedly increased (Fig. 4b, c). After L-arabinose treatment, the level of adiponectin increased by 22.2%, while leptin and TNF-α decreased by 11.1% and 13.6%, respectively. All of these differences were statistically significant.

The results of BUN and CK are showed in Table 5. The activities of BUN and CK in the metabolic syndrome group were increased by 54.34% and 16.47%, respectively compared to the Control group. After treatment with L-arabinose for 6 weeks, the activities reduced to 7.26+1.77 mmol/L and 223.52+16.85 mmol/L (Table 5) with statistically significant differences. These results showed that L-arabinose may protect the kidney and heart to a certain degree.

### The preliminary mechanism of L-arabinose on metabolic syndrome

#### Changes in the expression of adipose genes involved in inflammation

**Fig. 5:**
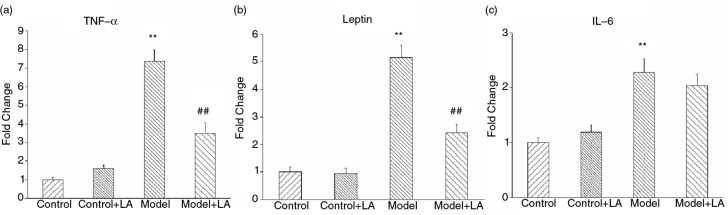
Effects of L-arabinose supplementation on the mRNA levels of inflammatory cytokines. (a) Gene expression of Adiponectin. (b) Gene expression of Leptin. (c) Gene expression of TNF-α. All values are the mean±SD. *p<0.05, **p<0.01 vs. Control; ^#^ *p*<0.05, ^##^ *p*<0.01 vs. Model.

In order to explore the possible mechanism of L-arabinose on metabolic syndrome, six target genes related to metabolism including TNF-α, leptin, IL-6, ACCα, CPT-1α, and CPT-1α were measured by real-time PCR in liver and adipose tissues. All adipose genes involved in inflammation were up-regulated. L-Arabinose did not demonstrate differences on mRNA expression between the Control+LA group and the Control group. The mRNA levels of TNF-α and leptin were decreased by approximately 52.6 and 51.9% in the Model+LA group compared to the Model group (Fig. 5a, b), respectively, while the expression level of IL-6 had no obvious change (Fig. 5c).

#### Molecular analysis of genes related to fatty acid metabolism induced by HCHF diet

**Fig. 6:**
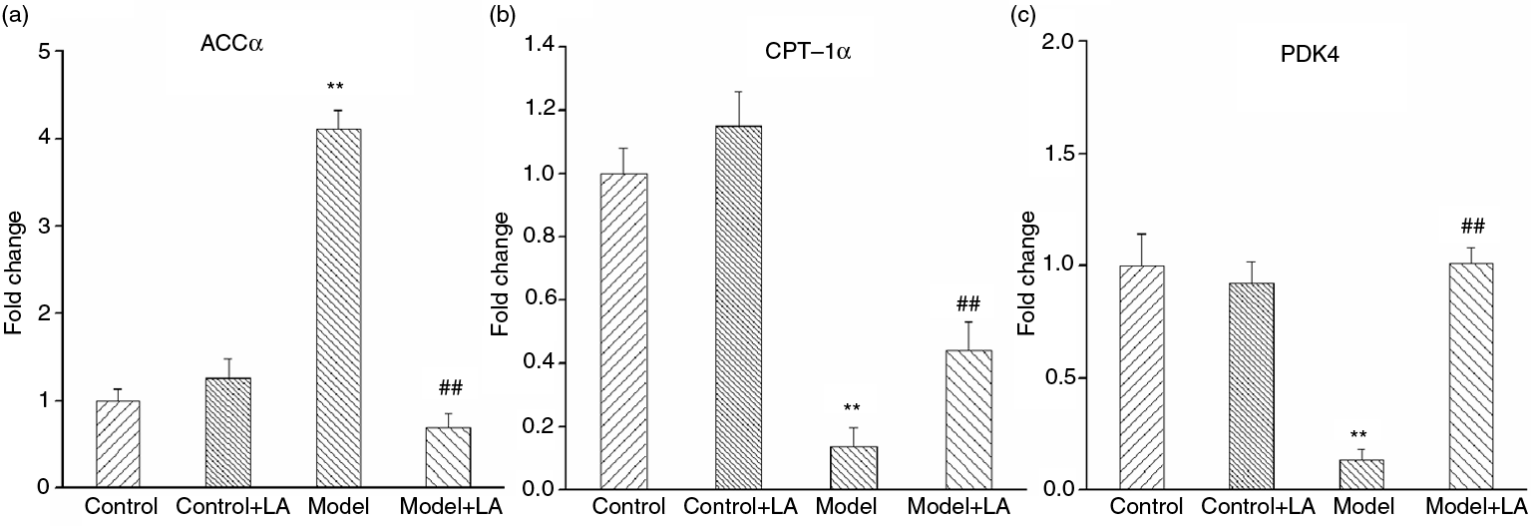
Changes in hepatic expression of genes involved in fatty acid metabolism and gluconeogenesis. (a) Gene expression of ACCα. (b) Gene expression of CPT-1α. (c) Gene expression of PDK4. All values are the mean±SD. *p<0.05, **p<0.01 vs. Control; ^#^ *p*<0.05, ^##^ *p*<0.01 vs. Model.

As shown in Fig. 6a, the mRNA levels of hepatic ACCα in the Model group are significantly higher than that of the Control group. Compared to the Model group, the mRNA levels of ACCα in Model+LA group rats returned to the normal level. CPT-1α is the rate-limiting enzyme of β-oxidation of fatty acids (14). The expression of CPT-1α was reduced by 86.1% in the Model group rats. Treatment with L-arabinose restored the decrease in CPT-1α expression level of by 30.2% (Fig. 6b). Gene expression levels of hepatic PDK4, the rate-limiting enzyme of glucose oxidation, were significantly decreased (87.0%) in the Model group. However, the changes were reversed to normal levels after L-arabinose treatment (Fig. 6c).

## Discussion

Metabolic syndrome is a cluster of dangerous factors for non-alcoholic fatty liver and cardiovascular diseases, including central obesity, dyslipidemia, hypertension, impaired glucose tolerance, and insulin resistance (15, 16). L-Arabinose is a pentose, produced by mild acid hydrolysis of sugar beets, and is absorbed from the intestinal tract in rats with a lower rate compared to glucose. Compared with other functional sugars, L-arabinose has its own advantages, such as a wide variety of sources, low sweetness, low cost, and so on. Furthermore, some human trails have proved that daily consumption of L-arabinose would inhibit the postprandial blood glucose level, insulin, C-peptide responses, and enhance the glucagon-like peptide-1 response, slimming down adults' body without gastrointestinal adverse effects as well (17, 18). Therefore, it is important to understand the effects of L-arabinose on the functioning of human organisms with chronic conditions, such as metabolic syndrome. In this study, we have estimated the efficacy of L-arabinose in rats fed with a HCHF diet as an appropriate model for human metabolic syndrome.

It had been reported that the dose of 1 g/kg L-arabinose did not cause diarrhea in rats and the LD50 value was 20 g/kg orally. The consumption of 2 g/day L-arabinose in adults (60 kg) does not cause any adverse effects on health (19). It has been suggested by the Food and Drug Administration that the extrapolation of animal dose to human dose should be performed through the body surface area comparisons. Based on it, the dosage for the rats in this study (400 mg/kg bodyweight/day) was calculated according to 2 g/day L-arabinose in adults using the formula according to the research of Reagan-Shaw (20). Doses of L-arabinose ranging from 50 mg/kg bodyweight to 1,000 mg/kg bodyweight have been used in several other reports (19, 21). In this study, the duration of the experiments (6 weeks) was chosen because some authors have found that several kinds of experimental materials, such as montelukast and irbesartan (22), can exhibit effects in diet-induced metabolic syndrome in rats after 6 weeks.

Evidence that links insulin resistance to the development of hypertension suggests that intervention aimed to create a state of hyperinsulinemia in experimental rats leads to elevated blood pressure. Furthermore, factors such as drug administration, physical training that enhanced insulin sensitivity, and reduced serum insulin levels also lower blood pressure (23). Treatment with L-arabinose has proven to cause a reduction of insulin concentrations and elevated insulin sensitivity in metabolic syndrome rats; thus, these changes could be partially responsible for the lowering effect on blood pressure. The reduction in serum TG, TC, and LDL-C levels and the increase in HDL-C, as well as the reduction of FFA in serum levels by L-arabinose have been reported by our studies and those of others (24). All these changes might play an important role in improving insulin sensitivity. It has been well reported that L-arabinose selectively inhibits intestinal sucrase activity in an uncompetitive manner by forming an enzyme-inhibitor-substrate (EIS) complex and suppresses the glycemic response (25, 26). While Jurgonski et al. believe that the suppression of postprandial glycaemia by L-arabinose in rats is more associated with starch than sucrose ingestion (27). In addition to the inhibition of digestive enzymes, L-arabinose also shows the protective effect on beta-cell, thus improving glucose tolerance in type-2 diabetic rats (21). These may all account for the mechanisms of L-arabinose on the reduction of insulin resistance. In our study, it was found that L-arabinose normalized the levels of GLUT4 (Table 4), which was responsible for glucose transport and uptake, and this also might be an important reason for alleviating insulin resistance by L-arabinose.

Excessive caloric intake induces visceral fat accumulation and further causes deregulation of adipocyte functions, including production of inflammation and oxygen free radicals (28). Inflammation exerted significant effects on the development of insulin resistance, obesity, and impaired glucose tolerance (29). At the white adipose tissue level, TNF-α has been shown to be over-expressed and was considered to be a linker between inflammation and metabolic syndrome. Other adipose-specific molecules that are related to energy metabolism also adjust immune responses (30). These data show that L-arabinose has strong anti-inflammation effects and effectively alleviates metabolic syndrome by changing the plasma levels and the expression of pro-inflammatory cytokines in adipose tissues.

It is necessary to evaluate the degree of damage to the liver, kidney, and heart in the metabolic syndrome rats. The liver, kidney, and heart were damaged to some extent in rats fed with a HCHF diet. However, L-arabinose supplement improved the degree of viscera injury, suggesting that L-arabinose protects the viscera state of metabolic syndrome rats. The related metabolism needs to be further studied.

Lipogenesis plays a critical role in hepatic lipid metabolism and is regulated by SREBP-1c (31). Excessive calorie uptake induced the expression of SREBP-1c, which was mediated by ACCα and FAS. Our results indicate that the mRNA expression level of ACCα in liver is significantly increased in the metabolic syndrome model. However, L-arabinose reverses the change, suggesting that L-arabinose improves hyperlipidemia by down-regulating ACCα.

PPARγ in hepatic tissue plays a key role in the development of hepatic steatosis (32). Hepatic lipid oxidation is another important factor in the development of fatty liver. CPT-1α is the rate-limiting enzyme associated with β-oxidation of fatty acids. As previously reported, the disturbance in β-oxidation of fatty acids owing to down-regulation of hepatic CPT-1α is a key mechanism in pathogenesis of fatty liver in metabolic syndrome (33, 34). Therefore, it was reasonable to propose that the reduction of hepatic lipid oxidation mediated by CPT-1α might be another important mechanism of hyperlipidemia in the metabolic syndrome model. In our study, treatment with L-arabinose led to down-regulation of expression of glucose oxidation enzymes that are related to hepatic glucose levels. PDK4 is the rate-limiting enzyme of glucose oxidation, and its expression was significantly decreased by 87.2% in the Model group. However, this change was reversed to normal levels after L-arabinose treatment. These results suggest that L-arabinose plays an indirect role in lowering blood glucose concentration.

## Conclusions

This work suggests that L-arabinose has an protective effect against the development of metabolic syndrome. It could lower body weight, Lee's index, visceral index, and SBP and improve dyslipidemia, insulin resistance, inflammation, and viscera function. These findings indicate that L-arabinose might be a promising candidate for therapies combating metabolic syndrome. Considering the preliminary stage of this study, further research is needed to clarify the multiple mechanisms of L-arabinose effects on metabolic syndrome.
